# Factors influencing the attitudes of NICU physicians toward care of neonates with very poor prognosis

**Published:** 2019-06-11

**Authors:** Fatemeh Nayeri, Fariba Asghari, Ali Baser, Leila Janani, Mamak Shariat, Kiarash Tanha, Bita Eabrhim

**Affiliations:** 1 *Professor, Family Health Institute, Maternal, Fetal and Neonatal Research Center, Tehran University of Medical Sciences, Tehran, Iran. *; 2 *Associate Professor, Medical Ethics and History of Medicine Research Center, Tehran University of Medical Sciences, Tehran, Iran. *; 3 *Researcher, Department of Pediatric, Arak University of Medical Sciences, Arak, Iran.*; 4 *Assistant Professor, Department of Biostatistics, School of Public Health, Iran University of Medical Sciences, Tehran, Iran.*; 5 *MSc, School of Public Health, Iran University of Medical Sciences, Tehran, Iran.*; 6 *Researcher, Family Health Institute, Breastfeeding Research Center, Tehran University of Medical Sciences, Tehran, Iran.*

**Keywords:** Invasive procedures, Newborns, Neonatal intensive care units, Prognosis, Resuscitation, Viability

## Abstract

Attitudes of physicians toward neonates with poor prognosis greatly influence their decisions regarding the course of treatment and care. The present study aimed to investigate factors contributing to attitudes of medical practitioners toward poor prognosis neonates. This was a cross-sectional, descriptive-analytic study. Questionnaires for assessing subjects’ attitudes toward care of very poor prognosis neonates were administered to all neonatologists, pediatricians, neonatology assistants, and pediatric residents (a total of 88 individuals) working in the NICUs of Imam Khomeini Hospital. Participants’ attitudes were determined through analysis of responses to seven questions on a 5-point Likert scale ranging from “strongly agree” to “strongly disagree”.

Presence of anomalies incompatible with an acceptable quality of life, birth weight, gestational age, responses to neonatal diagnostic tests, certain types of diseases, parental marital status and practitioner predictions about patient prognosis were the factors contributing to practitioners’ attitude (*P*-value < 0.005). However, no significant relationship was found in connection with religious beliefs, socioeconomic status, opinions of consulting physicians, hospital treatment protocols, standards of the Association of Neonatal Physicians, and ethics committee expectations (*P*-value > 0.005). It can be concluded that the attitudes of practitioners toward intensive care of poor prognosis neonates is determined by the medical condition of the neonate rather than socio-demographic characteristics.

## Introduction

The increase and ease of access to new fertility techniques coincide with an increase in the number of newborn infants requiring special care (1). Pregnancies resulting from such techniques are at risk of prematurity, low birth weight and multiple births, and are predisposed to a greater need for resuscitation notwithstanding other factors at birth (2). Preterm neonates, especially moderately preterm neonates (MPT, 29 - 33^6/7^ weeks), are more likely to have higher morbidity rates. Studies have shown higher severity of respiratory problems, cognitive disorders, and reduced academic and behavioral performance in children resulting from these pregnancies (3, 4). Furthermore, with the proliferation of Neonatal Intensive Care Units (NICU) and improvement of nursing practices and practitioner knowledge, it is now possible to preserve the lives of critically ill and severely premature infants. Thus, neonatologists are facing new issues such as the necessity for providing aggressive and advanced care to neonates with severely poor prognosis. Severe prematurity, advanced asphyxia, congenital anomalies incompatible with life, and advanced congenital metabolic disorders are examples of such cases.

There is a lack of specific regulations within national laws and the medical code of ethics in Iran regarding whether to initiate, continue or abandon advanced treatments. In some countries, the sanctity of life is a priority and it is therefore mandatory to employ any possible measure for every infant regardless of disease or prognosis (5). Other countries, however, support quality of life, allowing refusal of advanced treatment in cases of undesirably low levels of life quality (6). Still, others exist on a cline between these two extremes, making decisions based on their own moral and ideological principles as well as departmental policies (7, 8). In some countries, laws have been enacted whereby practitioners and the families involved can mutually decide on whether to continue advanced treatment (9). According to Rabiu and Sugand, English laws’ tolerance of quality of life judgments infringes on the sanctity of life doctrine, and hence does not allow this doctrine to be considered a law (10).

Considering the lack of a relevant set of national guidelines or laws in Iran, practitioners may decide based on religious or moral beliefs, hospital policies, or consultation with parents. 

In many cases, the practitioner is faced with the question of the determining criteria for continuing advanced treatment. One question that may arise is whether continuation of treatment will lead to loss and waste of resources in the society. Additional questions include: Would this not be in contradiction with commitment to medical justice in the society? Is allocating part of the limited resources of a society to an individual with a poor prognosis not tantamount to denying a greater number of individuals the access to those same limited resources? Regardless, in most cases, medical practitioners are burdened with a great responsibility in their decisions. Personal values and beliefs (in other words, the practitioner’s attitude) might play a fundamental role in decision-making and answering the aforementioned questions. 

Considering the fact that the number of NICUs is increasing by the day in Iran, more physicians encounter ethical challenges when facing critically ill infants. Practitioners’ decisions regarding continuation or discontinuation of treatment in incurable cases is of crucial importance, since there is a possibility of treating future infants requiring specialized care with the same attitude. The present study was carried out on medical practitioners employed in the fields related to neonatal medicine and childcare in the NICUs affiliated with Tehran University of Medical Sciences. The aim of this study was to investigate attitudes, as well as the factors influencing these attitudes, toward critically ill infants and those with very poor prognoses.

## Method

This was a cross-sectional, descriptive-analytic study conducted from January 2015 to June 2015 (5 months). The study population consisted of all neonatologists, pediatricians, neonatology fellows and pediatric residents working in 9 teaching NICUs affiliated with Tehran University of Medical Sciences. These intensive care units had 6 to 30 active beds and 26 to 50 admissions per month. After receiving the approval of the ethics committee, participants were asked to complete a questionnaire consisting of the Raines’ questionnaire titled “Level of Management in Neonatal Clinical Situations” (11) and Rebaglioto’s questionnaire (12), translated and merged. 

The questionnaires were translated and then approved by five experienced neonatologists based on measurement instruments of clinical ethics. Subsequently, they were tested on 20 cases and then used (Cronbach’s alpha 75%).

The final questionnaire consisted of 3 parts:

First, participants filled out a demographic questionnaire including their age, gender, marital status, number of children, health status of children, level of education, and work experience.

Next, three different cases of newborns suffering from severe prematurity, genetic disorder (chromosomal trisomy), and severe asphyxia were presented to participants. It was explained to the subjects that aggressive care treatment was the option for these three cases. The details of the treatment option were described to consist of aggressive care, including all necessary, practical measures that had to be taken to preserve the life of the infants (e.g. mechanical ventilation and invasive procedures). 

The participants were then asked, “Which of the following factors (and to what extent) affect your attitude and decisions regarding aggressive care of critically ill infants with poor prognosis?” The responses included gestational age, infant weight, parents’ marital status, parents’ social and financial status, infant’s disease, results of infant diagnostic tests, practitioner predictions regarding patient prognosis, presence of anomalies incompatible with an acceptable quality of life, opinions of consulting physicians, hospital treatment protocols, standards of the Association of Neonatal Physicians, ethics committee expectations, and moral and religious beliefs. 

Finally seven 5- point Likert scaled questions was used for the evaluation of respondents’ pro-life attitude. The pro-life beliefs questions are presented in Table 3. In order to evaluate respondents’ pro-life attitude, seven 5-point Likert scaled questions ranging from “strongly agree” to “strongly disagree” were used. The pro-life attitude of participants was defined as the sum of the points attributed to responses to these questions. Since there were seven questions, the range of scores varied between 7 and 35. 

It should be noted that in rating points, the highest score was given to "strongly agree" and the lowest score to the "strongly disagree" option. 

As for the concepts of questions, the first four questions and question number 6 highlighted the importance of sanctity of life, whereas numbers 5 and 7 had reversed concepts. 

With respect to the categorization of the answers, there were two opposing groups: the "agree" and "strongly agree" options were regarded as pro-life, while "neutral ", "disagree", and "strongly disagree" responses comprised the negative opinions. The seven questions and analysis of the responses are presented in Table 3.

The questionnaire was anonymous and did not require the participants to take any responsibility for the care of infants, or to cover any expenses. Furthermore, the study did not involve the treatment of infants. The study was approved (as a subspecialty thesis) by the Research Council of Tehran University of Medical Sciences. 

The new version of the Statistical Package for Social Sciences (SPSS) is not readily available in Iran due to sanctions, so SPSS version 11 was used for statistical analysis. To describe demographic data, descriptive statistics (i.e. frequency, mean, and standard deviation) were used. Frequencies (percentages) and mean (SD) were utilized to report categorical variables and continuous variables. Chi-square and Fisher’s exact tests compared the intergroup distribution of qualitative variables. In order to determine the association between variables, logistic regression was utilized. The significance level was considered as *P*-values bellow 0.05. The continuous variables were analyzed by one-way ANOVA, with a *P*-value bellow 0.05 designated as the statistically significant level.

## Results

Eighty-eight physicians from 9 different medical centers participated in the study. Participants' characteristics are presented in Table 1.

**Table 1:** Characteristics of the study population

		No. (Percent)
Gender	Male	61 (69.3)
	Female	27 (30.7)
Age	< 30	17 (20)
	30 - 45	60 (70.6)
	> 45	11 (9.4)
Marital Status	Married	70 (80)
	Single	18 (20)
Children (married subjects)	Yes	50 (71.4)
	No	20 (28.6)
Profession	Pediatric resident	45 (51.1)
	Pediatrician	23 (26.1)
	Fellow of neonatology	10 (11.4)
	Neonatologist	10 (11.4)
Work Experience (years)	< 5	42 (47.8)
	> 5	46 (52.2)

Twenty-seven of the responding physicians (35.1%) supported the use of aggressive measures in scenarios involving neonates with poor prognosis. Factors contributing to practitioners’ attitude toward aggressive care and participants’ decision about aggressive care in poor prognosis infant scenarios are presented in Table 2.

**Table 2:** Factors contributing to practitioners' attitude toward aggressive care

		Physicians’ Agreement with Aggressive Care		
Factors Influencing Physicians’ Decision on Aggressive Care		***Disagree*** ***No. (percent)***	***Agree*** ***No. (percent)***	***P-Value***
Gestational Age	Disagree	12 (24.5)	37 (75.5)	0.011
	Agree	14 (53.8)	12 (46.2)	
Birth Weight	Disagree	14 (28.6)	35 (71.4)	0.031
	Agree	14 (53.8)	12 (46.2)	
Parents’ Marital Status	Disagree	31 (64.6)	17 (35.4)	0.033
	Agree	22 (88.00)	3 (12.00)	
Socioeconomic Status	Disagree	35 (72.9)	13 (27.1)	0.253
	Agree	22 (84.6)	4 (15.4)	
Type of Disease	Disagree	3 (6.3)	45 (93.8)	< 0.001
	Agree	17 (68.00)	8 (32.00)	
Diagnostic Tests	Disagree	10 (20.4)	39 (79.6)	< 0.001
	Agree	18 (69.2)	8 (30.8)	
Prognosis	Disagree	11 (22.9)	37 (77.1)	< 0.001
	Agree	17 (68.00)	8 (32.00)	
Anomalies Incompatible with an Acceptable Quality of Life	Disagree	10 (20.4)	39 (79.6)	0.033
	Agree	11 (44.0)	14 (56.00)	
Consulting Physician’s Opinion	Disagree	11 (22.9)	37 (77.1)	0.062
	Agree	11 (44.0)	14 (56.00)	
Therapeutic Protocols	Disagree	30 (62.5)	18 (37.5)	0.686
	Agree	11 (42.3)	15 (57.7)	
Neonatology Association	Disagree	17 (36.2)	30 (63.8)	0.250
	Agree	13 (50.0)	13 (50.0)	
Ethics Committee	Disagree	33 (68.8)	15 (31.3)	0.280
	Agree	14 (56.00)	11 (44.00)	
Religious Beliefs	Disagree	29 (60.4)	19 (39.6)	0.136
	Agree	11 (42.3)	15 (57.7)	

Presence of anomalies incompatible with an acceptable quality of life, birth weight, gestational age, responses to neonatal diagnostic tests, type of disease, parental marital status and practitioner predictions about patient prognosis were factors contributing to practitioners’ attitude. However, no significant relationship was found in connection with religious beliefs, socioeconomic status, opinions of consulting physicians, hospital treatment protocols, standards of the Association of Neonatal Physicians, and ethics committee expectations.

Practitioners' attitudes toward aggressive care in terms of their pro-life beliefs are shown in Table 3, according to which the mean score was 20.96 ± 3.93 out of 35. The minimum score was 7 with the maximum being 27. The pro-life attitude scores of participants who agreed and those who disagreed with aggressive care were significantly different (*P*-value = 0.025). The mean attitude score of participants who agreed with aggressive care was 22/22 ± 3/96, and 20/18 ± 3/36 for those who disagreed.

The practitioners' attitudes toward aggressive care in terms of their pro-life beliefs are presented in Table 3.

**Table 3:** Practitioners' attitudes toward aggressive care in terms of their pro-life beliefs

Questions Used for the Evaluation of Respondents’ Pro-Life Attitude		Number and Valid Percent
**Due to the sanctity of life, every possible measure must be taken ** **to preserve the infant’s life, even in case of poor prognosis.**	Disagree	34 (43.6)
	Agree	44 (48.9)
**Even in the presence of severe physical disability, survival is ** **better than death.**	Disagree	56 (72.7)
	Agree	21 (27.3)
**Limiting special care in a specific case or select cases creates the ** **opportunity for exploitation, leading to more cases of ** **exploitation.**	Disagree	44 (57.1)
	Agree	33 (42.9)
**Every infant must be provided with the maximum level of care and ** **treatment without consideration of the end results, since such cases ** **can lead to improved treatment for future patients.**	Disagree	33 (42.9)
	Agree	44 (48.9)
**Increased therapy and rehabilitation costs for preterm infants and ** **disabled children do not give us the right to treat patients without ** **consideration of the end results.**	Disagree	36 (46.8)
	Agree	41 (53.2)
**My religious beliefs, especially in terms of Islamic justice, do not ** **allow me to discontinue special care in specific cases or consider ** **limitations in providing special care.**	Disagree	55 (71.4)
	Agree	22(28.6)
**My moral beliefs, especially in terms of preserving healthcare ** **justice, allow me to discontinue special care in specific cases or ** **consider limitations in providing such care.**	Disagree	51 (66.2)
	Agree	26 (33.8)

The evaluation showed no significant correlation between the socio-demographic characteristics of the practitioners and their decision regarding aggressive courses of treatment.

## Discussion

This study was conducted with the aim of investigating factors contributing to attitudes of neonatologists and pediatricians towards care for poor prognosis neonates in NICUs affiliated with Tehran University of Medical Sciences. Physicians’ attitudes toward sanctity or quality of life and factors contributing to such attitudes assume the main role in their decisions and performances in initiation, denial, and discontinuation of advanced treatment measures for infants with poor prognosis. 

In the Ghaffari et al. study, factors influencing practitioner decisions regarding discontinuation of resuscitation included the number of children in the infant's family, pressure on hospital employees, requests by the infant's parents, infant’s gender, insufficient hospital facilities or resources, history of infertility, financial status of the family, and religious beliefs (13). 

In the studies by Rebaglioto et al. and Cuttini et al. (2000 - 2006) in ten European countries, the following factors had an effect on attitude: greater belief in higher quality of life, being female, not having children, being a Protestant, lacking a religious background, disregard for religious beliefs, greater professional experience, and working in wards with higher admission of infants with very low birth weight (12, 14). 

Age, duration of work experience, and attention to religious beliefs were considered contributing factors in non-treatment decisions by physicians in the Cuttini et al study (15). In this study, 48.9% of physicians agreed that due to the sanctity of life, every measure must be taken to preserve the life of an infant, even with poor diagnosis. In Rebagliato et al study the views of physicians differed depending on the country where they worked (12). In Estonia and Lithuania, practitioners believed they had to implement any measure necessary for saving the patient regardless of the cost, whereas in Sweden and the United Kingdom, physicians’ decisions were based on quality of life. Physicians living in countries with higher socioeconomic status (such as Sweden and the United Kingdom) were more inclined toward quality of life. Conversely, in countries with lower socioeconomic states such as Lithuania, the view of sanctity of life held greater sway (12). In a coordinated manner, Romanian practitioners had no tendency to deny or discontinue special care for infants even with very poor prognoses (16).

In the present study, 72.7% of the physicians disagreed with the statement that despite any physical disability, survival is better than death. Thus, in this case they favored quality of life over the alternative. In a study of 10 European countries, presence of mental disability, as opposed to physical disability, had a greater effect on the attitudes of most physicians. Overall, most practitioners in various countries considered severe mental disability to be equivalent to or even worse than death, while there was less agreement on severe physical disability. Presence of mental disability had a greater effect on the attitudes of most physicians compared to physical disability in many countries (11). In the present study, however, mental disability was not specifically investigated.

It must be noted that despite evaluation of the correlation coefficient of the questions (0.613) as well as their conformity with each other, a conflict is observed in the responses of practitioners to the first two questions. This conflict is due to the fact that in the first question, the practitioners preferred to preserve the life when they were to choose between life or death. In the second question, however, among survival despite severe physical disabilities, multiple disabilities and death, they opted for death. 

In the present study, 57.1% of the physicians disagreed that limiting care in certain cases could lead to increased possibility of exploitation. A significant percentage of practitioners in Spain, Germany, Hungary, Lithuania, France and Italy agreed that treatment limitations might create an opportunity for exploitation. Many even believed that the reverse of this line of reasoning could also lead to maltreatment, specifically overtreatment. Most physicians agreed with a set of limitations in aggressive interventions due to very severe neurological prognosis (11).

In the present study, 48.9% of the physicians agreed that all infants must be provided with the maximum level of care due to the impact this may have on the treatment of future patients. In the EURONIC study, most practitioners claimed that they had encountered at least one situation where treatment measures were limited due to an incurable condition. In a few cases, treatment was limited due to poor neurological prognosis (15). In more than half of the countries in the Baltic region, legal restrictions prevent decisions to limit treatment, whereas in a low percentage of cases in Sweden (3%) and France (5%), it is possible to make decisions regarding limitation of treatment. A significant number of practitioners in Lithuania (54%) and Italy (29%) agreed with treating every infant regardless of the results, in order to gain clinical experience and benefit future generations (11).

On the other hand, 46.8% of the physicians in our study disagreed with the statement that increased cost of treatment is an acceptable reason to limit care for infants based on their prognosis. In the European study, most physicians did not believe that the costs of healthcare affected decisions for treatment. However, in France, the United Kingdom, and the Baltic states, a quarter or more agreed that such costs did in fact affect decisions. Furthermore, costs of healthcare and clinical experiences played a lesser role in decision-making for legislators in enacting legal restrictions (11). 

Moreover, 71.4% of the physicians in our study believed that religious beliefs should not affect the decision to continue or discontinue care, while 66.2% believed that the decision to discontinue care should not be based on moral beliefs. In a European study, the attitude scores regarding quality of life were significantly lower among physicians whose religious beliefs were important to them (Italy, Hungary, and the Baltic countries) compared to countries where religious beliefs were not as important (14). 

Cuttini et al. state that in some countries, limitations in special care exist due to moral reasons (15). However, identification of the conditions necessitating limitation of treatment and decisions that are made in such conditions differ in various countries based on culture. At the same time, however, legal considerations certainly have an influential role. In most countries, the decision to deny treatment to infants who will die despite medical intervention is acceptable. However, limiting special care based on future quality of life is still a controversial issue. In most countries, factors including practitioners’ religious beliefs and personal attitudes toward the value and meaning of life influence decision-making (14).

Zamboni expresses that neonatologists make their decisions based on their personal beliefs (17). In Italy, even though a physician is obligated to obey the professional and medical codes of ethics, which refute aggressive care, Italian law strongly supports preservation of the lives of infants and opposes any type of discrimination based on abnormalities or poor prognoses. In addition, the law requires resuscitation of a preterm infant even when birth is caused by a late abortion. Zamboni stresses the importance of accepting differences in decision-making since many disabilities may be accepted by the society, which may possess the moral progressiveness to support its weakest members (17). 

The American Academy of Pediatrics (AAP) supports abandonment of treatment when it is not in the infant’s favor. It allows deciding on discontinuing treatment for neonates and infants only in the following cases: (1) when the infant is in an irreversible coma; (2) when the treatment only prolongs the process of death; (3) when the treatment is futile and inhumane (18). 

The AAP supports joint decision-making with parents concerning resuscitation and intensive care of infants born at extremely low gestational age (< 25 weeks). In such cases, both the infant and the family face the possibility of permanent, severe neurodevelopmental risks and costly health care, and therefore measures to help the infant to merely “survive” will not be justifiable (18 - 19).

In conclusion, considering the results of the present study, attitudes towards intensive care of poor prognosis neonates are correlated with their medical conditions rather than socio-demographic characteristics. Furthermore, due to the lack of special regulations within national laws and the medical code of ethics, most practitioners are trapped in a sort of legal vacuum. Thus, it seems necessary to legislate laws that clearly determine cases requiring initial denial of treatment and the conditions for discontinuing such treatment. Additionally, the pain and suffering of infants need to be considered, and parents must be given sufficient privileges to determine the course of action that is in the best interests of their child.

## Notes:

**Citation to this article:**

Nayeri F, Asghari F, Baser A, Leila Janani L, Shariat M, Tanha K, Eabrhim B. Factors influencing the attitudes of NICU physicians toward care of neonates with very poor prognosis. J Med Ethics Hist. 2019; 12: 6.
